# Plasma metabolomics supports the use of long-duration cardiac arrest rodent model to study human disease by demonstrating similar metabolic alterations

**DOI:** 10.1038/s41598-020-76401-x

**Published:** 2020-11-12

**Authors:** Muhammad Shoaib, Rishabh C. Choudhary, Jaewoo Choi, Nancy Kim, Kei Hayashida, Tsukasa Yagi, Tai Yin, Mitsuaki Nishikimi, Jan F. Stevens, Lance B. Becker, Junhwan Kim

**Affiliations:** 1 Laboratory for Critical Care Physiology, Feinstein Institutes for Medical Research, 350 Community Dr, Manhasset, NY 11030 USA; 2 Donald and Barbara Zucker School of Medicine At Hofstra/Northwell, Hempstead, NY USA; 3 Linus Pauling Institute, Oregon State University, Corvallis, OR USA; 4 Department of Pharmaceutical Sciences, Oregon State University, Corvallis, OR USA; 5 Department of Emergency Medicine, Northwell Health, NY USA

**Keywords:** Biochemistry, Chemical biology

## Abstract

Cardiac arrest (CA) is a leading cause of death and there is a necessity for animal models that accurately represent human injury severity. We evaluated a rat model of severe CA injury by comparing plasma metabolic alterations to human patients. Plasma was obtained from adult human control and CA patients post-resuscitation, and from male Sprague–Dawley rats at baseline and after 20 min CA followed by 30 min cardiopulmonary bypass resuscitation. An untargeted metabolomics evaluation using UPLC-QTOF-MS/MS was performed for plasma metabolome comparison. Here we show the metabolic commonality between humans and our severe injury rat model, highlighting significant metabolic dysfunction as seen by similar alterations in (1) TCA cycle metabolites, (2) tryptophan and kynurenic acid metabolites, and (3) acylcarnitine, fatty acid, and phospholipid metabolites. With substantial interspecies metabolic similarity in post-resuscitation plasma, our long duration CA rat model metabolically replicates human disease and is a suitable model for translational CA research.

## Introduction

Cardiac arrest (CA) affects more than 400,000 people annually in the US^1,2^. Most cases that occur outside the hospital result in longer durations of ischemia and increased metabolic injury; during this time, the brain and other organs have been susceptible to metabolic dysregulation, resulting in low survival rates^3–6^. A recent study administered a mixture of metabolic and cytoprotective agents using an ex vivo perfusion system to potentiate metabolic support in post-mortem pig brains and identified decreased cellular death and retention of cytoarchitecture despite functional impairment of the brain^7^. With this complex disease process, a more sophisticated understanding of CA pathophysiology using a good representative preclinical model is required.

Most current animal models use short durations of CA^8–12^, which may be unable to produce a considerable ischemic or reperfusion injury to accurately represent the majority of human CA patients. Short duration CA animals are able to recover and survive for several days without much treatment^13,14^. However, most human patients suffer longer durations of CA and as a result, succumb to the severe injuries within a few days, thus requiring a long duration CA rat model. Since longer duration CA animals cannot be effectively resuscitated using conventional chest compressions, a customized cardiopulmonary bypass (CPB) system based on venous-arterial extracorporeal membrane oxygenation (ECMO) has been used for rodents^15,16^, which has been utilized to resuscitate humans who also do not respond to conventional CPR^17^. CPB enables consistent resuscitation even after 20 min of prolonged CA.

In this study, we applied a metabolomics approach to elucidate the metabolic comparability of the dysregulated metabolome of typical human CA patients with severe injury and our long duration CA rat model. Metabolomics enables accurate measurement of the metabolic status in humans with minimal invasiveness by quantitating metabolites that are not easily achieved with the use of the other traditional “-omics” technologies^18–20^. Our study measures metabolites from the plasma obtained from rats after 20 min asphyxia-induced CA followed by 30 min CPB resuscitation and compares it to plasma from human non-CA controls and CA patients. Here, we highlight key metabolite alterations and provide the global context in which that metabolic dysfunction occurs between both species in order to ascertain the degree of metabolic similarity.

Our data suggests that despite the variations of species, mode of resuscitation, and heterogeneity vs homogeneity of human and rodent CA, respectively, the metabolic dysfunction that occurs because of CA and resuscitation is exceedingly similar. This long duration of CA rodent model simulates a severe global injury that appears to represent the pathology observed in our human patients.

## Results

### Physiological outcomes

Patient samples were obtained from severe CA patients. All 13 patients died within 3 days, with only 3 patients surviving past 24 h. Ten patients had sustainable return of spontaneous circulation (ROSC), lasting more than 1 h. Supplementary Table 1 summarizes the various available patient characteristics. The outcomes of rats used for this study were similar to the human patients. The average survival time of rats after 20 min CA and 30 min CPB resuscitation was 3.8 ± 0.38 h with limited brain function as seen as a lack of response to toe pinch and corneal agitation. The average time to reach CA, which is mean arterial pressure below 20 mmHg was 186 ± 42 s. Approximately 45 ± 27 s after CPB initiation, cardiac electrical activity was observable, and initial ROSC was achieved on average in 200 ± 81 s. The long duration of CA produces a severe brain injury resulting in limited neurological function post-resuscitation and poor survival outcomes, which is the general trend observed in human CA patients.

### Principal component analyses of plasma metabolites for humans and rats after resuscitation

**Figure 1:**
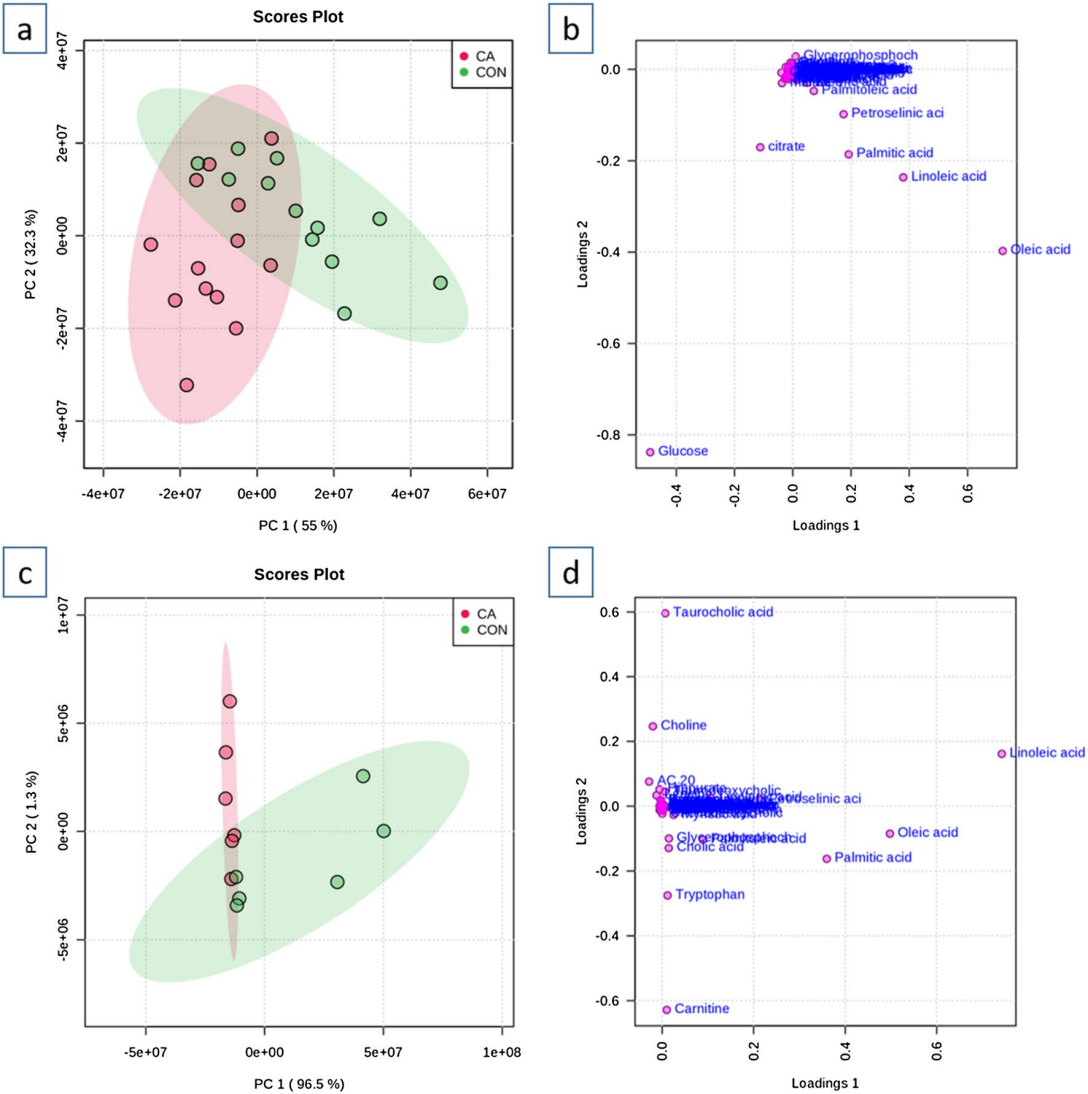
Principal Components Analysis of human and rat control and post-CA plasma. (**a**) Scores plot of metabolites from control and post-CA human plasma; (**b**) Loading plot of metabolites from control and post-CA human plasma; (**c**) Scores plot of metabolites from control and post-CA rat plasma; (**d**) Loading plot of metabolites from control and post-CA rat plasma. Although no complete separation was observed between control and post-CA plasma in both humans and rats, there is a noticeable metabolic difference that can be attributed to alterations in fatty acids, amino acids, and TCA metabolites as a function of CA and resuscitation. *CON* control, *CA* post-CA and resuscitation.

Principal component analysis (PCA) was performed to illustrate differences using 93 common metabolites detected in both humans and rats. All PCA plots have 95% confidence regions displayed for each group. The unbiased PCA analysis shows metabolites from human and rat control samples with a unique and distinct cluster as compared to the clustering of CA samples, confirming the sufficient sample size in both the species to conduct further analyses (Fig. 1). Furthermore, a supervised analysis, Sparse Partial Least Squares Discriminant Analysis (sPLSDA), also confirms this distinct clustering of CA and control metabolites with a similar trend in the metabolic divergence as a function of CA and resuscitation in both humans and rodents (Supplementary Fig. 1). The resulting similar pattern of metabolic alterations after CA compared with the respective controls of both humans and rats warrants a deeper examination of the metabolite profile post-CA.

The scores plot of human plasma shows PC1 and PC2 values of 55% and 32.3% of data variation, respectively, to show the metabolic changes between control and post-CA plasma. Despite some overlap with control plasma, the human post-CA metabolome shows a distinct global alteration, as observed by the deviation in the scores plot (Fig. 1a). The loading plots show the correlation in the various metabolites in the control and post-CA plasma for both humans and rats. The loading plot for human plasma shows free fatty acids, such as linoleic, oleic, and palmitic acids, with loading 1 greater than 0.2 and loading 2 of − 0.1 to − 0.4 producing the majority of variation; glucose and citrate also contribute to this variation with glucose contributing most of the metabolic variation with loading 1 of greater than − 0.4 and loading 2 of greater than − 0.8 (Fig. 1b). The scores plot for rat plasma shows PC1 and PC2 values of 96.5% and 1.3% of data variation, respectively, highlighting the metabolic variations between control and post-CA plasma (Fig. 1c).

The scores plot of the rodent plasma shows a similar metabolic dysfunction pattern as human plasma when compared with their respective controls produced by similar metabolite changes that can be seen in the loading plots for both humans and rats. The loading plot shows that the free fatty acids, such as linoleic, oleic, and palmitic acids with loading 1 of 0 to 0.8 and loading 2 of 0.2 to − 0.2 seem to account for the majority of variation between the control and post-resuscitation rat plasma (Fig. 1d). Carnitine, tryptophan, choline, and taurocholic acid also contribute to the metabolic variations with loading 2 ranging from − 0.6 to 0.6. The rodent analysis reveals that fatty acids, along with some bile acids and carnitine help to contribute to the metabolic alteration after resuscitation, while the human analysis reveals fatty acids, citrate, and glucose as the major contributors. Although no complete separation was observed between control and post-CA plasma in both humans and rats, there is a noticeable metabolic difference that can be attributed to post-CA alterations in fatty acids, amino acids, and TCA metabolites as a function of CA and resuscitation. Overall, both species show distinct clusters for control and post-CA plasma, which have a similar pattern of dysregulation meriting a deeper analysis.

### Changes in individual metabolites following cardiac arrest and resuscitation in humans and rats

**Figure 2:**
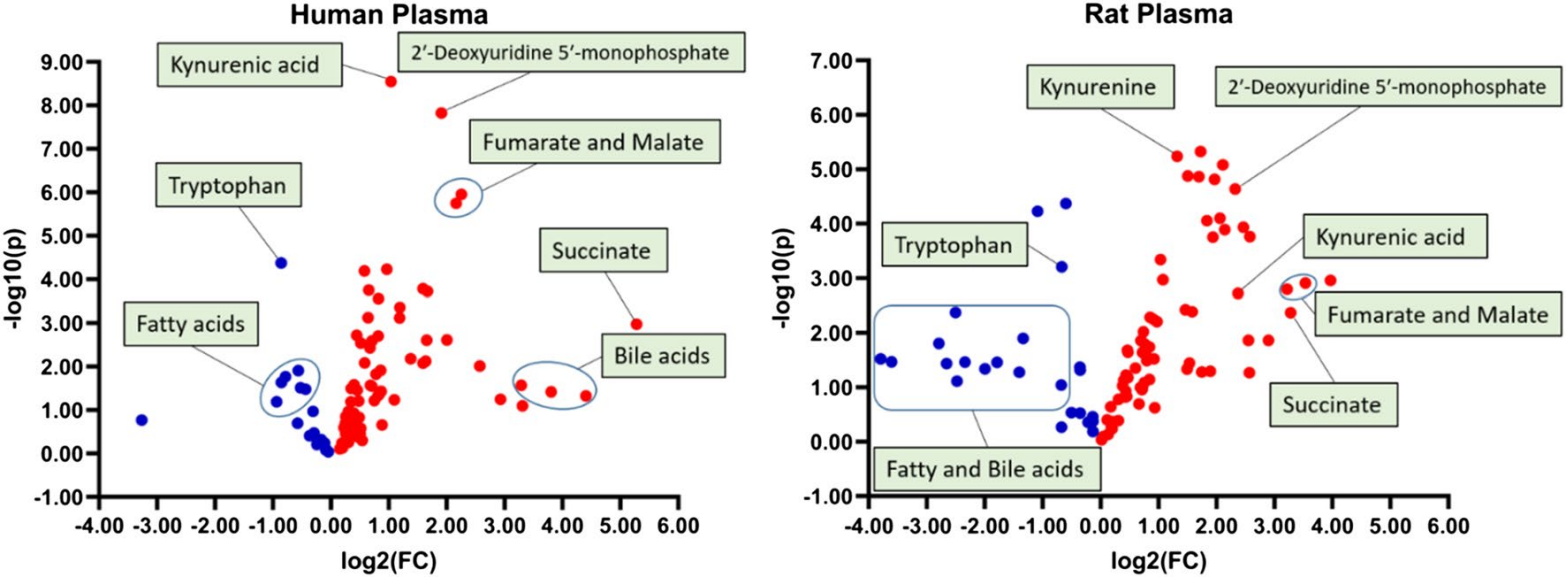
Metabolic disarray post-CA has a similar pattern in humans and rats. Volcano plot comparison of metabolites in human (left) and rat (right) plasma post-CA identifies substantial changes in metabolites via fold change. Red indicates increased levels, while blue indicates decreased levels.

The volcano plot aids in visualizing and identifying substantial changes in overall metabolites and determining trends in the overall metabolome alterations post-CA and resuscitation between humans and rats (Fig. 2). Metabolites with a − log10(p) value greater than 1.30 are statistically significant. Humans and rats have a similar overall distribution in altered metabolites, such that greater than 75% metabolites measured are either increased, or show an increasing trend, measured in terms of fold change from control. Kynurenic acid, 2′-Deoxyuridine 5′-monophosphate, fumarate, malate, and succinate are all substantially increased, while most of the fatty acids and tryptophan are decreased after resuscitation in humans and rats. Bile acids are found to be increased in humans, while decreased in rats after resuscitation.

**Figure 3:**
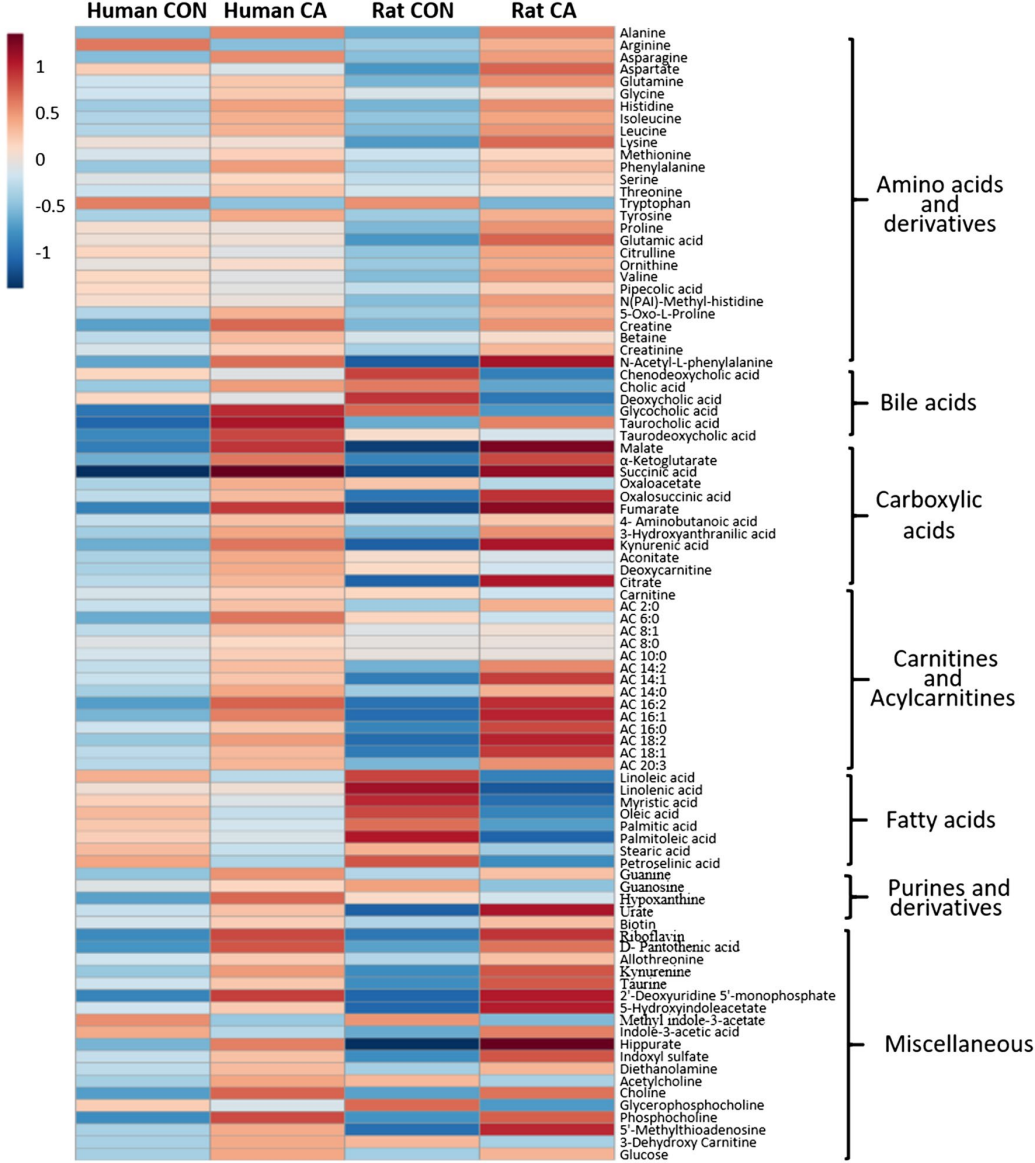
Humans and rats have similar metabolic alteration patterns post-CA. Heatmap depicts major metabolite alterations in human and rat plasma after CA compared to a normalized control. Metabolites represented as means are arranged in their corresponding metabolic group. The colors of each metabolite represent the degree of variation after resuscitation as compared to control: red indicates increased levels, while blue indicates decreased levels. *CON* control, *CA* post-CA and resuscitation.

Changes in individual metabolites are further presented in a heatmap, which can aid in visualizing the fold changes in each metabolite to show the patterns of alterations (Fig. 3). A simplified heatmap highlights only relative changes after resuscitation and very clearly illustrates a similar metabolic pattern (Supplementary Fig. 2). There were 28 amino acids and their derivatives measured of which 17 were increased in human plasma post-CA, such as alanine, asparagine, glutamine, glycine, histidine, isoleucine, leucine, methionine, phenylalanine, serine, threonine, tyrosine, 5-oxo-L-proline, creatine, betaine, creatinine, and *N*-acetyl-L-phenylalanine. There were 7 metabolites that decreased, such as arginine, aspartate, tryptophan, citrulline, valine, pipecolic acid, and *N*-(Pai)-methyl-L-histidine. In rat plasma, all measured amino acids and their derivatives were increased after CA and resuscitation except for tryptophan, which was substantially decreased.

There were 6 bile acid metabolites measured in humans and rats of which cholic, glycocholic, taurocholic, and taurodeoxycholic acids were increased in humans after CA. Rat plasma post-CA showed an increase in taurocholic acid and a decrease in chenodeoxycholic, cholic, deoxycholic, and glycocholic acids.

Out of the 12 carboxylic acid metabolites measured, all were found to be increased in humans post-CA, while only 9 were increased in rats post-CA, such as malate, α-ketoglutarate, succinate, oxalosuccinic acid, fumarate, 4-aminobutanoic acid, 3-hydroxyanthranilic acid, kynurenic acid, and citrate. In the rat plasma, 3 metabolites, oxaloacetate, aconitate, and deoxycarnitine, were found to be decreased. From the carboxylic acid metabolites measured, 9 metabolites were commonly increased in both humans and rats after CA and resuscitation were malate, α-ketoglutarate, succinate, oxalosuccinic acid, fumarate, 4-aminobutanoic acid, 3-hydroxyanthranilic acid, kynurenic acid, and citrate. Most of these metabolites are found in the TCA cycle.

There were 15 acylcarnitine (AC) species that were compared between human and rat plasma post-CA. Human plasma indicates all metabolites were increased, such as carnitine, AC2:0, AC6:0, AC8:1, AC8:0, AC10:0, AC14:2, AC14:1, AC14:0, AC16:2, AC16:1, AC16:0, AC18:2, AC18:1, and AC20:3. Similarly, 12 acylcarnitine species were increased in the rat plasma, such as AC2:0, AC8:1, AC8:0, AC14:2, AC14:1, AC14:0, AC16:2, AC16:1, AC16:0, AC18:2, AC18:1, and AC20:3. Acylcarnitines with longer fatty acid chains tend to be increased in both human and rat plasma after CA (Supplementary Fig. 3). Out of the 8 measured free fatty acids, 7 of them, linoleic, myristic, oleic, palmitic, palmitoleic, stearic, and petroselinic acids, were found to be decreased in human plasma and all 8 fatty acids were found to be decreased in rat plasma post-CA. There is significant homology between human and rat plasma in terms of acylcarnitine species and fatty acids after CA and resuscitation.

Purine metabolites that were measured show an increase in guanine, guanosine, hypoxanthine, and urate in humans after resuscitation, while only guanine and urate were increased and guanosine and hypoxanthine were decreased in rat plasma. Other analyzed metabolites that did not directly correspond with the available categories or whose related metabolites were unable to be measured were organized in the miscellaneous group. The measured B vitamins, biotin, riboflavin, and D-pantothenic acid showed the same trend after CA in both humans and rats, with riboflavin and D-pantothenic acid showing an increase post-CA. Allothreonine, kynurenine, taurine, 2′-deoxyuridine 5′-monophosphate, 5-hydroxyindoleacetate, hippurate, indoxyl sulfate, diethanolamine, choline, phosphocholine, 5′-methylthioadenosine, and glucose were all increased in both humans and rats post-CA. In humans, 2 metabolites, such as indole-3 acetic acid and methyl indole-3 acetate, were decreased, while rat plasma showed a decrease in 3-dehydroxycarnitine, methyl indole-3 acetate, acetylcholine, and glycerophosphocholine.

Overall, the majority of metabolites measured showed a similar alteration pattern between humans and rats as a function of CA and resuscitation. As such, it is imperative to discern the metabolic alterations in the context of well-accepted metabolic pathways, which can be depicted as energy-generating metabolites of TCA cycle, amino and fatty acids, as well as kynurenine and acylcarnitine metabolites^21,22^.

### Changes in plasma citric acid cycle metabolites following CA and resuscitation

**Figure 4:**
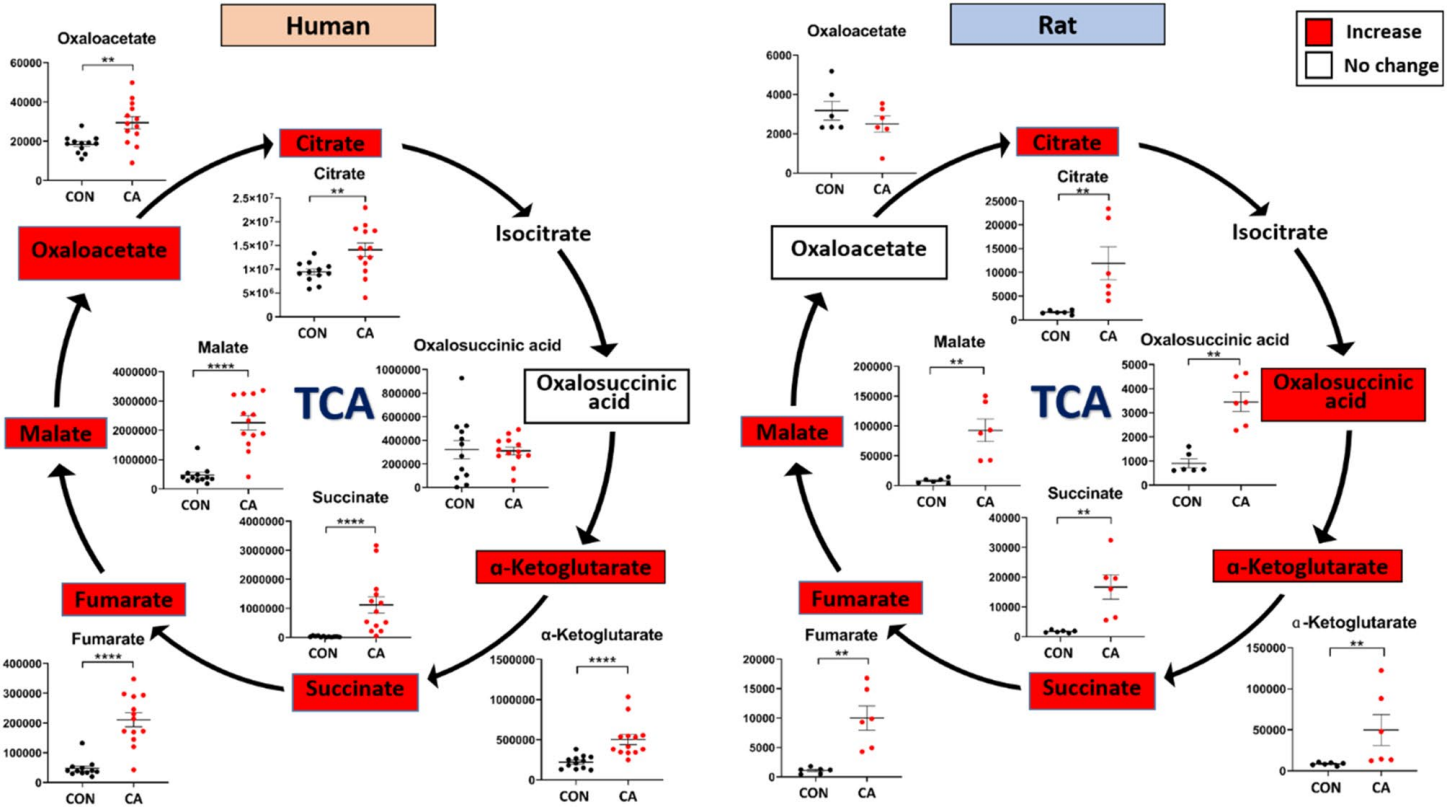
Highly similar TCA metabolite dysregulation post-CA in both humans and rats. Major metabolite alterations in the TCA cycle of human (left) and rat (right) plasma after CA. The colors of each metabolite correspond to the variation of that metabolite after resuscitation as compared to control: red indicates increased levels, while white indicates no change in levels. Both the human and rat plasma shows a substantial increase in almost all TCA metabolites post-resuscitation. *CON* control, *CA* post-CA and resuscitation, *y-axis* peak area. Data are displayed as mean ± SEM. Statistical analyses for each metabolite were performed using Mann–Whitney U test; **P < 0.01, ***P < 0.001, and ****P < 0.0001.

The citric acid cycle, also known as the TCA cycle, is the focal point of energy generation during aerobic respiration and its alterations have been discussed in the context of ischemic injury. Our data suggests that after CA and resuscitation, TCA cycle metabolites were significantly affected in both humans and rats, as determined by measuring plasma metabolites (Fig. 4). Citrate, α-ketoglutarate, succinate, fumarate, and malate were all significantly increased after resuscitation in both humans and rats when compared with their respective controls. Although the overwhelming metabolic similarity between the two species is apparent, the minor difference in TCA metabolite alterations is seen in oxaloacetic acid and oxalosuccinic acid of which the former is increased significantly in humans, while the latter is increased significantly in rats post-CA.

**Figure 5:**
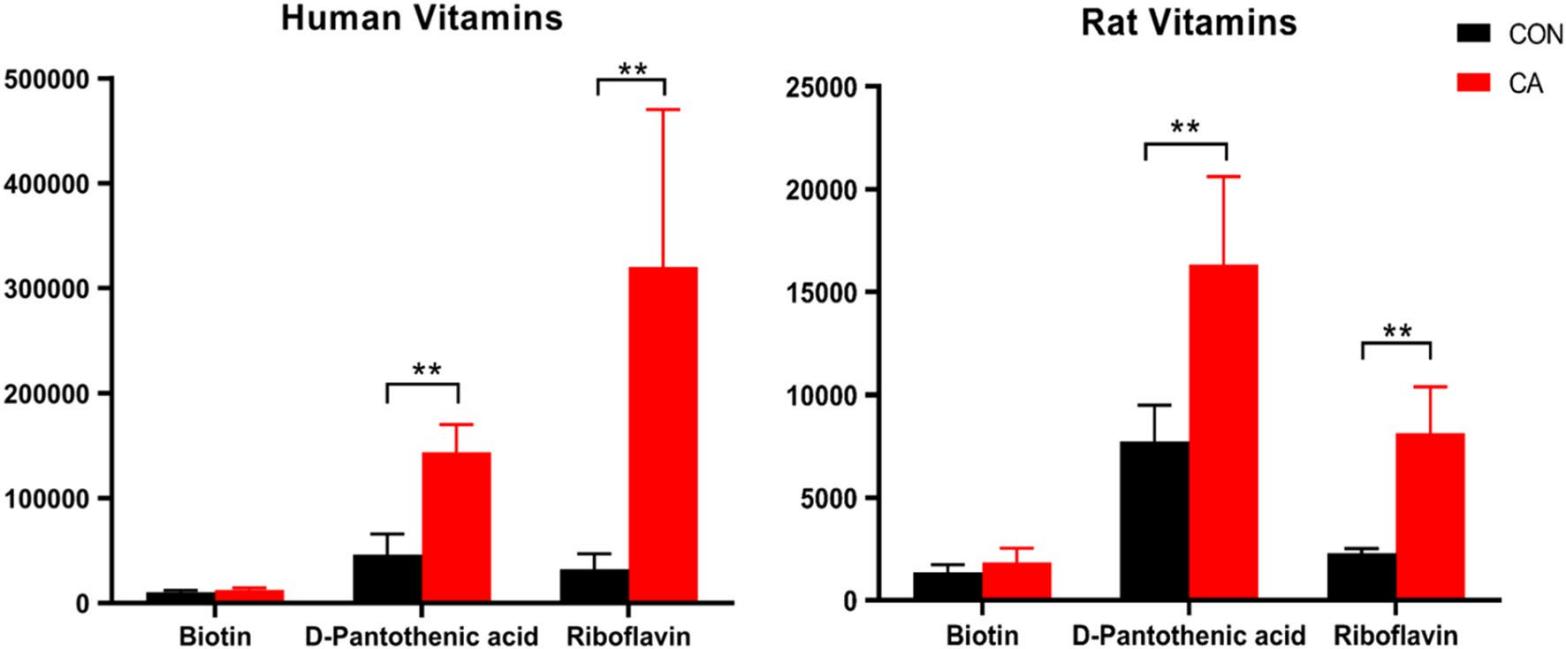
Similar vitamin alterations post-CA. Alterations in three water-soluble, B vitamins in humans (left) and rats (right) after CA as compared with their respective controls. Both human and rat plasma show a substantial increase in the levels of D-pantothenic acid and riboflavin after CA. *CON* control, *CA* post-CA and resuscitation, *y-axis* peak area. Data are displayed as mean ± SEM. Statistical analyses for each metabolite were performed using Mann–Whitney U test; **P < 0.01.

Furthermore, the same pattern was observed in three of the measured vitamins that function as enzyme cofactors in certain pathways of energy generation in tandem with some of the glycolytic and TCA cycle metabolites, among other functions. Both D-pantothenic acid and riboflavin significantly increased after resuscitation, while biotin remained mostly unchanged in both humans and rats when compared to their respective controls (Fig. 5). In short, humans and rats experience a severe dysregulation in almost all TCA metabolites and some vitamin cofactors post-CA, which can be readily detected in the plasma.

### Changes in plasma kynurenic acid pathway metabolites following CA and resuscitation

**Figure 6:**
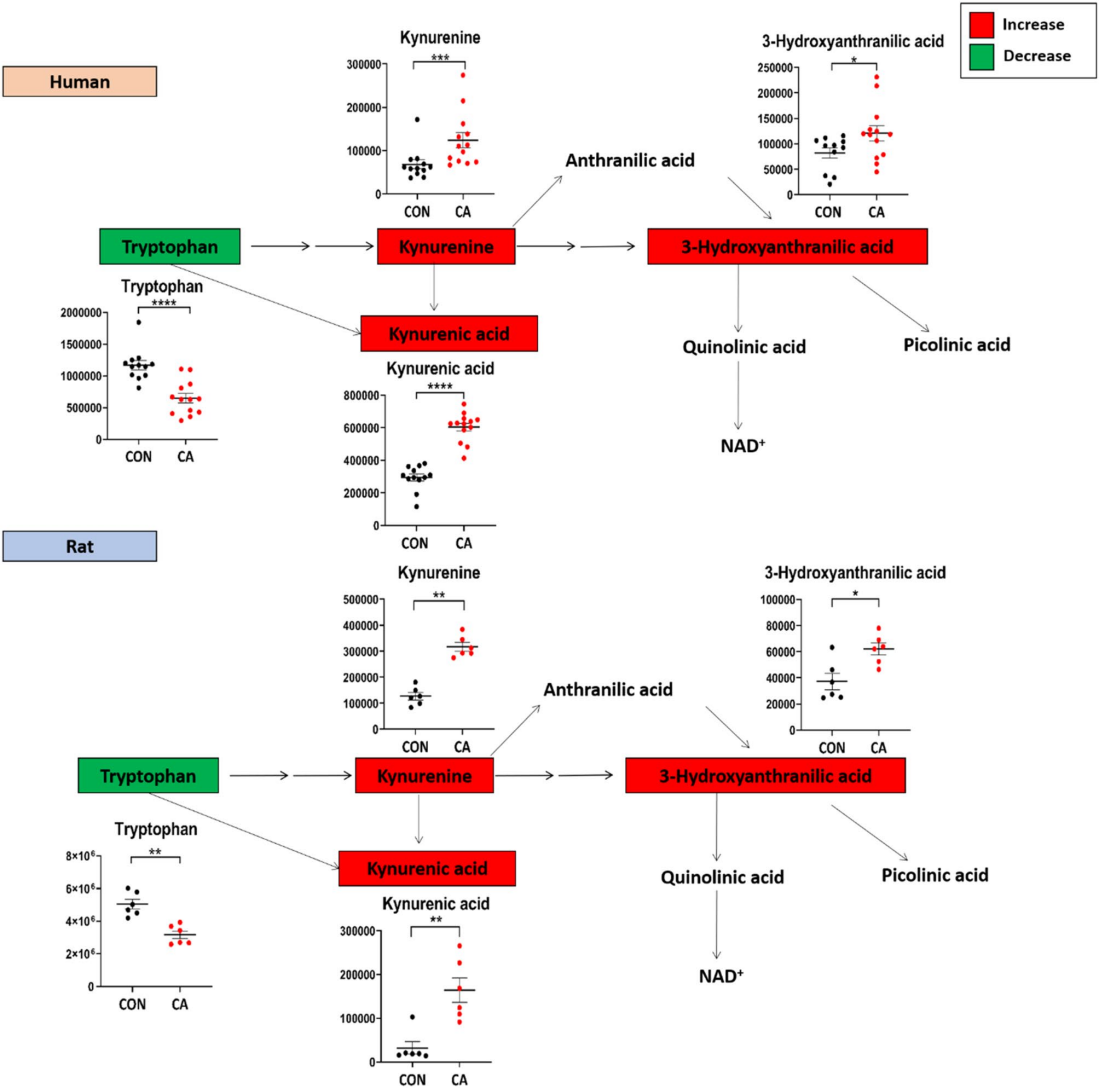
Highly dysregulated kynurenic acid pathway metabolites post-CA reveals similar dysfunction in humans and rats. Plasma kynurenic acid pathway metabolite alterations in human (top) and rat (bottom) plasma after CA. The colors of each metabolite correspond to the variation of that metabolite after resuscitation as compared to control: red indicates increased levels, while green indicates decreased levels. Both human and rat plasma show a substantial increase in most kynurenic acid pathway metabolites and a substantial decrease in tryptophan levels. *CON* control, *CA* post-CA and resuscitation, *y-axis* peak area. Data are displayed as mean ± SEM. Statistical analyses for each metabolite were performed using Mann–Whitney U test; *P < 0.05, **P < 0.01, ***P < 0.001, and ****P < 0.0001.

The alterations in the plasma levels of tryptophan and kynurenic acid pathway metabolites are markedly similar in both humans and rats. CA and resuscitation resulted in a significant decrease in tryptophan and a significant increase in kynurenine, kynurenic acid, and 3-hydroxyanthranilic acid in both humans and rats as compared to their respective controls (Fig. 6). Other downstream metabolites of this pathway, such as quinolinic acid and picolinic acid were not detected in the plasma.

### Changes in bile acids following CA and resuscitation

Bile acids are derivatives of cholesterol that are produced by various enzymatic reactions in the liver, while secondary bile acids are produced by the intestinal flora, both of which can subsequently be conjugated with glycine and or taurine to form conjugated bile acids^23^.

**Figure 7:**
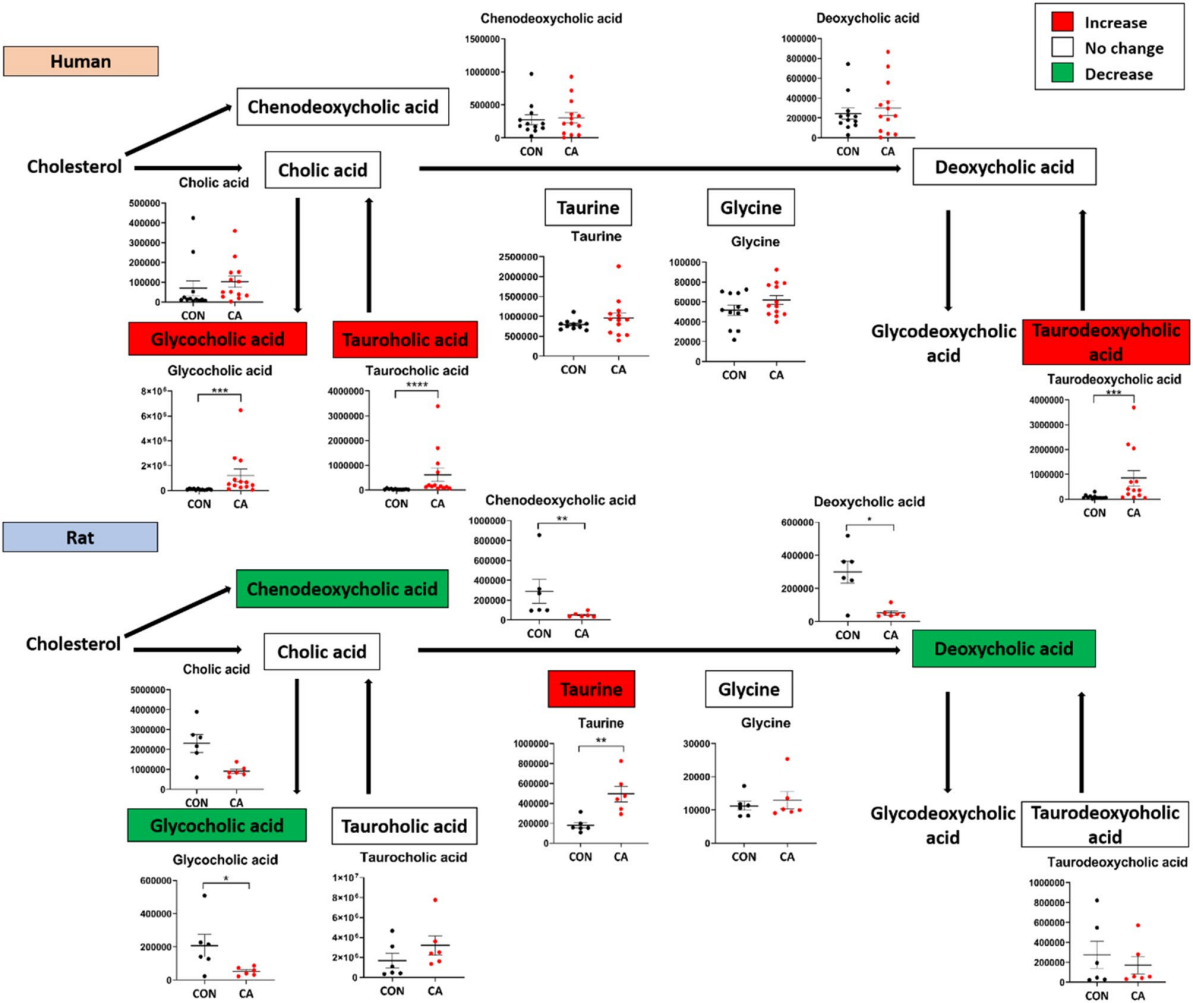
Bile acid metabolites reveal less similarity in post-CA alterations in humans and rats. Bile acid metabolite alterations in human (top) and rat (bottom) plasma after CA. The colors of each metabolite correspond to the variation of that metabolite after resuscitation as compared to control: red indicates increased levels, green indicates decreased levels, while white indicates no change in levels. The bile acid levels in humans and rats show some dissimilarity after resuscitation, such that a general increase in the conjugated bile acids, particularly the taurine conjugated acids, was observed in human plasma, while a general decreasing trend was observed in the rats with the exception of taurine. *CON* control, *CA* post-CA and resuscitation, *y-axis* peak area. Data are displayed as mean ± SEM. Statistical analyses for each metabolite were performed using Mann–Whitney U test; *P < 0.05, **P < 0.01, ***P < 0.001, and ****P < 0.0001.

The major changes in human plasma bile acids after resuscitation were observed in conjugated bile acids, such as glycocholic, taurocholic, and taurodeoxycholic acids, which all significantly increased when compared to control (Fig. 7). Deoxycholic acid, taurine, and glycine did not change significantly after CA and resuscitation. The primary bile acid, chenodeoxycholic acid, as well as the secondary bile acid, deoxycholic acid, and the conjugated bile acid, glycocholic acid, all significantly decreased after CA and resuscitation when compared to control in the rat plasma. The levels of taurine were significantly increased after arrest in the rats. Overall, the bile acid levels in the humans and rodents were not directly similar after resuscitation, such that a general increasing trend in the measured conjugated bile acids was observed in the humans, while a general decreasing trend was observed in the rats.

### Changes in plasma phospholipid metabolites and free fatty acids following CA and resuscitation

**Figure 8:**
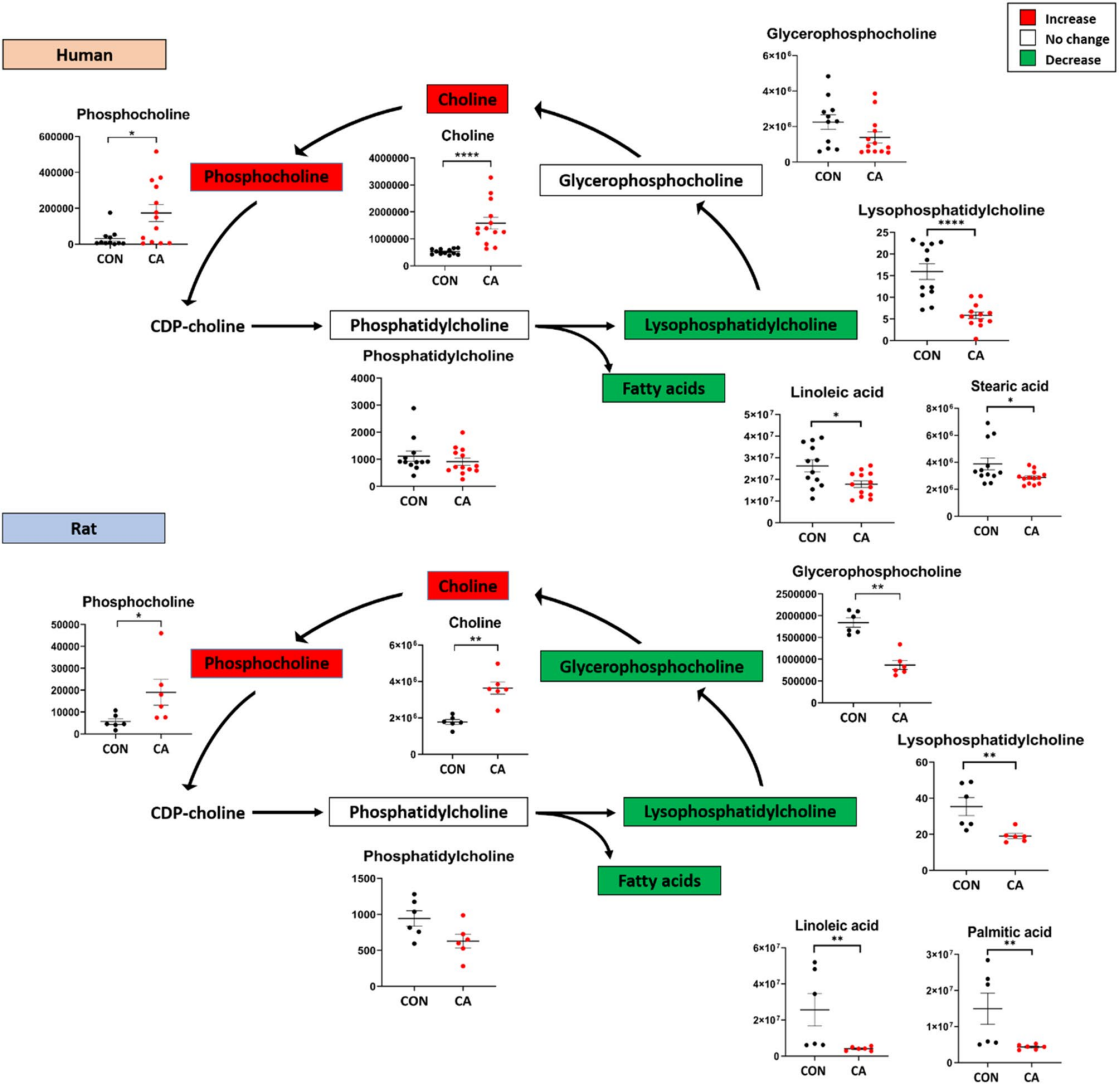
Choline and Phospholipid metabolites have similar alterations post-CA. Choline metabolite alterations in human (top) and rat (bottom) plasma after CA. The colors of each metabolite correspond to the variation of that metabolite after resuscitation as compared to control: red indicates increased levels, green indicates decreased levels, while white indicates no change in levels. Both human and rat plasma post-CA display a substantial increase in choline and phosphocholine, while showing a decrease in certain fatty acids. *CON* control, *CA* post-CA and resuscitation, *y-axis* peak area. Data are displayed as mean ± SEM. Statistical analyses for each metabolite were performed using Mann–Whitney U test; *P < 0.05, **P < 0.01, ***P < 0.001, and ****P < 0.0001.

Choline and phospholipid metabolites are important biochemical molecules that serve as major components of biological membrane and function as signaling molecules, among other roles^24^. After CA and resuscitation, plasma levels of choline and phosphocholine significantly increased in both humans and rats as compared to their respective controls, while the levels of lysophosphatidylcholine decreased in both species (Fig. 8). The levels of various fatty acids that are the constituents of phospholipids were similarly altered in both humans and rats (Supplementary Fig. 4). Glycerophospholipid significantly decreased in rat plasma and showed a decreasing trend in human plasma post-CA. The alterations of phospholipid precursors were readily detectable in both human and rat plasma post-CA and resuscitation and displayed a similar pattern of dysfunction.

## Discussion

CA pathophysiology comprises of substantial metabolic dysfunction resulting from ischemic damage and reperfusion injury^25,26^. Rodents have overwhelmingly modeled human CA, but generally experience shorter duration CA resulting in an injury that inefficiently simulates human CA injury^27,28^. Consequently, we utilized a rodent model of 20 min CA followed by CPB resuscitation to better replicate human injury severity. Here, we applied a metabolomics approach to evaluate the metabolic comparability of this severe injury model as a surrogate of human disease and identify key metabolic alterations to further characterize CA pathology. Despite the contrasting species, modes of resuscitation, and experimental regulations of the human and rodent CA environment, the metabolic dysfunction accompanying CA and resuscitation is exceedingly similar. The post-resuscitation plasma metabolome in both species exhibits similar characteristics: (1) an increase in TCA cycle metabolites, (2) severe alterations in the tryptophan and kynurenic acid pathway, and (3) severe alterations in the phospholipid pathway, decreased free fatty acids, and increased longer-chain acylcarnitine species. Overall, the current study is the first to utilize metabolomics to directly compare the metabolic alterations in the plasma of human CA patients and a severe rat model of CA; our data indicates that the major metabolic dysfunction is attributed to CA pathology, surpassing the differences in species and modes of resuscitation, and supporting the use of this model for the translation of metabolic attributes of this disease.

Mitochondrial injury in post-CA tissues is one major mechanism for organ damage that can result in death^29–33^ with only certain organs having the potential for improvement post-resuscitation^22,34^. Our current data illustrates that metabolite alterations attributed to tissue dysfunction post-CA are observed as metabolite derangements in the plasma (Figs. 4, 5, 6, 7, 8). Recent metabolomics evidence suggests plasma accumulation of non-lactate metabolites post-hypoxia is an important contributor to post-ischemic pathophysiology^35,36^. A few metabolomics studies in rodents^37^ and pigs^38,39^ established metabolic signatures in experimental models of milder injury CA, such as alterations in urea and TCA cycle metabolites, and anaplerotic metabolites of the TCA cycle^39^ in the plasma, a similar finding to a model of severe hepatic ischemia–reperfusion injury^40^. Our study corroborates the metabolic damage shown in the literature as seen as anaplerotic substrate and main TCA metabolite alterations (Fig. 4). A common metabolic change emphasized in the context of CA and cardiac ischemia is the increase of succinate^39,41,42^ that is also indisputable in our study of human and rat plasma (Fig. 4). The focus on one or a few TCA metabolites as the epitome of ischemia–reperfusion dysfunction^35,39,40,43^ is too simplified and does not precisely capture the complexity of CA. The interrelationship of succinate and other metabolite accumulation denotes diminished TCA cycle function in the mitochondria of various tissues that contributes to the plasma metabolite pool. In fact, identified B-vitamins, such as D-pantothenic acid and riboflavin that function as enzyme support, were also severely increased in the plasma indicating inappropriate energy metabolite physiology (Fig. 5). As such, the use of individual metabolites as proxy of disease severity has some relevance but does not completely depict the entirety of the post-CA metabolic disarray.

Additionally, we observed significantly increased longer-chain acylcarnitine species in the plasma, which reflect mitochondrial levels of acyl-coenzyme A species that are energy generating intermediates of fatty acids oxidized in the mitochondria^44,45^ (Supplementary Fig. 3). A substantial increase of acylcarnitine levels in the plasma signifies a loss of mitochondrial integrity in individual tissues for fatty acid oxidation^46^, resulting in incomplete β-oxidation of fatty acids whose product appear as medium-chain acylcarnitines in the plasma. The perturbation of both the TCA and the acylcarnitine metabolites post-CA suggest a dysfunctional mitochondrial status in the tissues that can be reflected in the plasma.

A mild form arrhythmia-induced CA in rats identified decreased plasma levels of tryptophan and increased levels of kynurenine, kynurenic acid, and 3-hydroxyanthranilic acid in the initial 2 h after resuscitation, with only increased kynurenic acid and 3-hydroxyanthranilic acid observed in pigs 1 h post-resuscitation^37^. This supports the association of alterations in the kynurenine pathway metabolites with worse outcomes in humans post-CA^37,47^. Our findings corroborate these reports from independent investigators regarding post-CA alterations in the tryptophan and kynurenine metabolism (Fig. 6). However, these striking similarities were observed within the first hour in humans and first half hour in rats after resuscitation, indicating that unlike previous studies, alterations in the tryptophan and kynurenine pathway metabolites occur almost immediately post-resuscitation. A higher kynurenine to tryptophan ratio observed in both preclinical and clinical studies of brain injury^48–51^ and cardiovascular disease^52^ indicated worse clinical outcomes implicating oxidative and inflammatory stress. Controversially, ischemic damage induced by CA may increase kynurenic acid, which may serve a protective role, as kynurenic acid has neuroprotective effects, while 3-hydroxyanthranilic acid and downstream quinolinic acid have a proclivity for neurotoxicity^50,53^. Although downstream metabolites were unable to be measured, substantial plasma alterations of this pathway suggest an immediate metabolic dysfunction in many organ systems, which invokes the application of inhibitors of this pathway to mitigate kynurenine metabolism into more cytotoxic metabolites^50,53–55^.

Although most of the identified metabolites revealed significant similarity between humans and rats post-CA, bile acids do not directly align with this pattern (Fig. 7). The overall differential reliance of these cholesterol derivatives between the two species may potentially explain this observation. Rodents tend to conjugate bile acids more frequently using taurine, while humans do so with glycine^23^, suggesting that slight changes in the minor species may seem more substantial than changes in the major species. Additionally, the different modes of resuscitation along with the species and intestinal flora differences may also contribute to this result. Bile acids function as emulsifiers to help digest and uptake lipids and fat-soluble vitamins, but also serve as signaling molecules for modulating metabolism and inflammation^56^. High levels of bile acids interfere with blood brain barrier integrity and act as direct cytotoxic agents^23,57,58^. It is possible that CA-induced global injury and the resulting diminished physiologic and metabolic processes may result in altered bile acid concentration in the plasma, which may be a potential mediator of reperfusion injury. Bile acid sequestration or use of a modified anti-inflammatory and cytoprotective bile acid, may counteract the detrimental effects of the increased bile acids^59–61^.

Our long duration CA rat model reveals that free fatty acids and lysophosphatidylcholine are significantly altered in the reperfusion phase, mirroring human injury (Fig. 8 and Supplementary Fig. 4). The downstream metabolites of phospholipids, choline, and phosphocholine, were significantly increased, while lysophosphatidylcholine and fatty acids were decreased. These are major biomolecules involved in signaling, membrane support and stability, and metabolism^24,62,63^. Resuscitation induces an increased metabolic shift towards phospholipid constituents, implicating an imbalance between the synthesis and metabolism of certain phospholipids and potential downstream destabilization of tissue cell membranes. Recently, studies are beginning to examine the role of phospholipids and other lipid dysregulation after CA^16,64,65^.

A limitation in our study is that the metabolomics comparison is based on the commonly detected metabolites, which does not encompass the entirety of the metabolome. Furthermore, the quantitation in our untargeted analysis is based on peak areas rather than absolute quantitation, and future studies will focus more on absolute quantitative change in metabolites, especially when examining certain interventions and targeting specific metabolic pathways. Despite these limitations and the differences in species, modes of resuscitation, and CA environment, the highly similar plasma metabolomics profile in humans and rats indicates that CA and resuscitation pathology is the primary mediator of these metabolic alterations. Our global metabolomics analysis provides the closest link to cellular metabolism in the whole body and its response to CA injury, which supports this model as a tool to help facilitate future studies for translation to human patients.

Taken together, our interspecies untargeted metabolomics analysis demonstrates the novel finding that after resuscitation, the metabolic milieu of the plasma in human CA patients is substantially similar to that of the rodent model of severe CA. Our data demonstrates that the variations in individual metabolites are suggestive of mitochondrial dysfunction post-CA, as seen by alterations in TCA cycle metabolites, and longer-chain acylcarnitine species in various tissues as reflected in the plasma. We also observed similar alterations in the kynurenic acid pathway and phospholipid metabolites in both humans and rats that provide a new avenue for exploration in CA. The substantial metabolic homology between humans and a severe CA injury rat model suggests that CA-driven metabolic dysregulation has a similar physiologic pattern in both species and that this is a suitable model for representing human CA.

## Methods

### Chemicals and materials

Chemical reagents utilized for metabolomics analyses were LC–MS grade methanol, acetonitrile, and ultra-pure water purchased from EMD Millipore (Gibbstown, NJ, USA); formic acid, acetic acid, ammonium hydroxide purchased from Fisher Chemical (Pittsburgh, PA, USA); and ammonium carbonate purchased from Sigma Aldrich (St. Louis, MO, USA).

### Animals and surgical procedures

The experimental protocol was approved by the Institutional Animal Care and Use Committee of the Feinstein Institutes for Medical Research. All procedures were conducted following the approved protocol. Adult male Sprague–Dawley rats (weight 410–450 g, Charles River Production, Wilmington, MA) were used for this study. Rats were maintained under a 12-h light/dark cycle with free access to food and water and used without starvation. Rats (n = 18, 6 in each group and 6 rats as blood donor) were arbitrarily assigned into two groups, control and 20 min CA with 30 min resuscitation via CPB. The established asphyxia-induced CA rat model was used as previously published^16,66^. Briefly, rats were anesthetized with 4% isoflurane and ventilated after intubation with 2% isoflurane. After the injection of heparin (300 IU) and vecuronium (1 mg), asphyxia was induced by stopping the ventilator and discontinuing isoflurane. Following 20 min of asphyxia, resuscitation was started with the initiation of CPB flow and reinitiating the ventilator. All rats achieved return of spontaneous circulation in ~ 3.5 min after the initiation of resuscitation. Rats were euthanized by decapitation 30 min after CPB was initiated after receiving 20 min of asphyxia prior; control group animals were euthanized after 7 min of isoflurane to ensure deep anesthesia. Blood samples were collected from control and after 20 min of asphyxia with 30 min of CPB resuscitation. The collected blood was centrifuged to separate plasma, which was stored at − 80 °C until analyzed.

### Human patient sample collection

For the human study, all procedures were conducted in accordance with the protocols approved by the Institutional Review Board at the Feinstein Institutes for Medical Research (protocol number 17-0030). Informed consent was waived by the Institutional Reviewer Board to collect blood samples and de-identified patients’ information. Blood samples were collected from 12 non-CA control and 13 CA patients admitted to the emergency department of North Shore University Hospital with out-of-hospital CA and achieved return of spontaneous circulation (ROSC) within 1 h after blood draw. The samples were deidentified to protect patient identity; the researchers analyzing the blood sample for metabolites were blinded to the patient and clinical history of the CA and prognosis. The basic patient characteristics available are shown in Supplementary Table 1. Whole blood was centrifuged using benchtop centrifugation (1000 *g*, 10 min at 4 °C) to separate plasma from red blood cells. Plasma was stored at − 80 °C until analysis.

### Sample extraction from human and rat plasma

The plasma samples were thawed on ice, then 100 μL of plasma was transferred to 1.5 mL Eppendorf tubes. For deproteinization and extraction for metabolomics analyses, solvent (400 μL, 50:50, v/v, methanol:acetonitrile), was added, vortexed for 30 s, incubated for 1 h at − 20 °C, then centrifuged at 4 °C at 15,000 × *g* for 10 min. Aliquots (400 μL) of the upper layer were transferred individually to new tubes and dried by nitrogen gas steam. The dried extract was reconstituted in solvent (200 μL, 50:50 v/v acetonitrile:water), vortexed for 20 s, centrifuged at 4 °C at 15,000 × *g* for 5 min, and then stored at − 80 °C until analysis via LC–MS/MS. To ensure the stability and repeatability of the LC–MS system, quality control (QC) samples, which consist of an equal mixture of 10 μL aliquots of representative biological samples from the same study; after every four samples, QC was ran so that the analytical variation is much smaller than the biological variability. The coefficient of variation is usually within 15% when the signal-to-noise ratio is > 10. To measure phosphatidylcholine and lysophosphatidylcholine, 50 μL of plasma samples were extracted with 750 μl of methanol as previously described^67^. Samples were randomized and human samples were run in one batch and rat samples in another. The run time for each batch for each column was less than 24 h and the autosampler was maintained at 4 °C during the run.

### Metabolomics analysis

Metabolomics analysis was performed using a previously established method^22^. Briefly, the metabolomics analysis, ultra-high performance liquid chromatography (Shimadzu Nexera system; Shimadzu; Columbia, MD) coupled to a high resolution hybrid quadrupole-time-of-fight (TOF) mass spectrometer (MS) (TripleTOF 5600; Sciex; Framingham, MA) was utilized. Every sample underwent analysis via two different methods using either reverse phase and HILIC columns in both the negative and positive ion modes^21^. High performance liquid chromatography (Agilent 1100 Series) coupled to LTQ-XL (Thermo Fisher) was used to analyze plasma levels of phosphatidylcholine and lysophosphatidylcholine^67^. Peak area of each metabolite, measured within the linear range of the mass detector, was normalized to plasma volume for each sample and presented as mean ± SEM.

### Data processing

Data was processed during analysis using a previously established method^22^. Briefly, untargeted metabolomics data was processed using Peak View 2.1 and MultiQuant software version 3.0.2 (SCIEX). The acquired mass data were analyzed using PeakView with XIC Manager 1.2.0 (Sciex, Framingham, MA) for peak picking, retention time correction, and peak alignment. Metabolite identities were assigned by matching accurate mass (error < 10 ppm), retention time (error < 10%), MS/MS fragmentation (library score > 70), and isotope distribution (error < 20%) with an in-house library consisting of IROA standards (IROA Technology, Bolton, MA) and other commercially available standards (650 total). In addition to the IROA database, the fragmentation spectra of all peaks were verified with data from Metlin and HMDB. Only one missing value was observed in the human samples, which was dropped after verification of the peak intensity below the limit of quantitation.

### Multivariate and statistical analyses

A total of 125 and 100 metabolites were detected in human and rat plasma, respectively, with 93 metabolites that were common to both species that were used for this comparison study. Principal component analysis (PCA) was used to examine the metabolic profile of human and rat plasma to display the overall changes after the ischemia–reperfusion injury that follows CA and resuscitation compared to their respective controls. To further analyze the changing pattern in metabolites to facilitate comparison between humans and rats post-CA, a volcano plot and heatmap were generated. The PCA, Sparse Partial Least Squares Discriminant Analysis (sPLSDA), volcano plot, and heatmap were all generated using MetaboAnalyst (v4.0). The heatmap was generated by normalizing the data with log transformation and pareto scaling. Statistical analyses for each metabolite were performed using Mann–Whitney U test, using GraphPad Prism 8.0 software (GraphPad, La Jolla, CA). Alterations in various metabolites as compared to control were mapped and diagrammed in well-recognized biochemical pathways as previously published^22^.

## Supplementary information

Supplementary Information

## Data Availability

The data that support the findings of this study is available from the corresponding author (JK) upon reasonable request.
