# Supplementary material for: Plasma metabolomics supports the use of long-duration cardiac arrest rodent model to study human disease by demonstrating similar metabolic alterations
Source: Sci Rep. 2020 Nov 12;10:19707. doi: 10.1038/s41598-020-76401-x (PMC7665036; doi:10.1038/s41598-020-76401-x)
Supplement: Supplementary file 1 — Supplementary Information [file 41598_2020_76401_MOESM1_ESM.pdf]

Supporting Information to

Plasma metabolomics supports the use of long-duration cardiac arrest rodent model to study human disease by demonstrating similar metabolic alterations

Muhammad Shoaib[1,2,+], Rishabh C. Choudhary[1,+], Jaewoo Choi[3,4], Nancy Kim[2], Kei Hayashida[1], Tsukasa Yagi[1], Tai Yin[1], Mitsuaki Nishikimi[1], Jan F. Stevens[3,4], Lance B. Becker[1,2,5], and Junhwan Kim[1,2]*

1. Laboratory for Critical Care Physiology, Feinstein Institutes for Medical Research, Manhasset, NY, USA
2. Donald and Barbara Zucker School of Medicine at Hofstra/Northwell, Hempstead, NY, USA
3. Linus Pauling Institute, Oregon State University, Corvallis, OR, USA
4. Department of Pharmaceutical Sciences, Oregon State University, Corvallis, OR, USA
5. Department of Emergency Medicine, Northwell Health, NY, USA

+ Both authors contributed equally

* Correspondence to: Junhwan Kim, Ph.D.

Laboratory for Critical Care Physiology

The Feinstein Institute for Medical Research

350 Community Dr.

Manhasset, NY 11030

Phone: 1-516-562-0452

Email: jkim46@northwell.edu

Supplementary Methods:

Metabolomics analysis:

For each sample, two different analysis methods were performed using reverse-phase and hydrophilic interaction LC columns. In reverse-phase LC, chromatographic separations were carried out on a 2.1×100 mm Titan C18 column (1.9 μm, Supelco, Bellefonte, PA) for positive and negative ion analyses. The sample injection volume was 5 μL, and the flow rate was 0.2 mL/min. The mobile phases consisted of water (A) and methanol (B), both with 0.1% formic acid. The gradient was as follows: an initial hold at 3% B for 0.5 minutes, followed by a gradient of 3% to 20% B in 5 minutes, to 55% B in 13 minutes, to 95% B in 15.5 minutes, held until 18.5 minutes, then a shift to 5% B at 19 minutes until 25 minutes. The column temperature was held at 45°C. In metabolomics hydrophilic interaction LC analysis, separation was carried out on a 2.1×150 mm SeQuant-ZIC-pHILIC (5 μm, EMD Millipore, Billerica, MA)[21]. The flow rate was 0.2 mL/min and the injection volume was 5 μL. The two mobile phases, water (A) and acetonitrile (B), consisted of 20 mmol/L ammonium carbonate, and were adjusted to pH 9.2 with a 25%-30% solution of ammonia in water. The gradient was as follows: an initial hold at 80% B for 2 minutes, followed by a gradient of 80% to 20% B in 17 minutes, then a shift to 80% B at 17.1 minutes until 22 minutes. The column temperature was 45°C.

Time-of-flight-MS was operated with an acquisition time of 0.25 seconds and a scan range of 70 to 1000 m/z. MS/MS acquisition was performed with collision energy set at 35 V and collision energy spread of 15 V. Each MS/MS scan had an accumulation time of 0.12 seconds and a range of 40 to 1000 m/z using information-dependent acquisition (IDA). The source temperature was set at 500°C in reverse phase and at 550°C in hydrophilic interaction LC. The IonSpray voltage at 5.5 kV in positive ion mode and −4.5 kV negative ion mode.

Phosphatidylcholine and lysophosphatidylcholine analysis:

The HPLC system (Agilent 1100 Series) to separate each class of phospholipid was developed by modification of a previously reported method[69]. A Nucleosil diol column (5 µm, 3×250 mm) from Macherey-Nagel (Duren, Germany) was used. Eluent A contained IPA:TBME:ammonium formate (340:170:50) and eluent B contained MeOH. Aqueous ammonium formate (pH ~2.5) was prepared by dissolving 295 mg of ammonium formate and 2 mL of formic acid in 50 mL of water. The gradients used for the 40 min chromatographic analysis were as follows: 100 % A for 20 min, 100% A to 20% A over 6 min, 20% A for 6 min, 20% A to 100% A over 1 min, and hold 100% A for 7 min. The flow rate was 0.3 mL/min and the column temperature was 30°C. MS data were obtained with a Thermo LTQ XL mass spectrometer operated in the negative ion mode, collecting full scan MS data from 180 – 2000 m/z. The source parameters were sheath gas flow (8 units), spray voltage (4 kV), capillary temperature (300 °C), and capillary voltage (-9 V).

Supplementary Table 1. Human patient characteristics comparing control patients and post-CA and resuscitation patients. There were no statistical differences between the two groups in the measured characteristics. CA: CA; ROSC: return of spontaneous circulation.

**Characteristics of control and CA patients**	**Control (n=12)**	**CA patients (n=13)**	**P value**
Gender-Male (%)	6 (50.0 %)	6 (46.2 %)	0.848
Mean Age (range)	74.1 (62-87)	73.6 (59-92)	0.922
Sustainable ROSC	N/A	10 (76.9 %)	-
Survival 1 day or more (%)	N/A	3 (23.1 %)	-
Survival to discharge (%)	N/A	0 (0%)	-

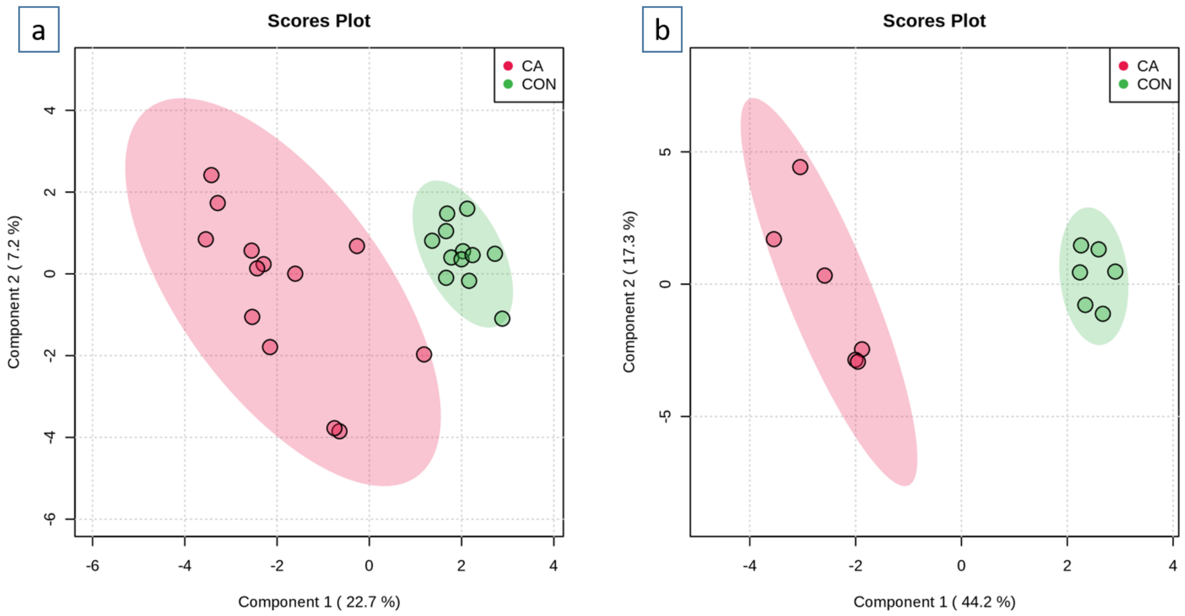

Supplementary Fig. 1. **Supervised PCA analysis reveals similar metabolic dysfunction pattern post-CA in humans and rats.** Sparse Partial Least Squares Discriminant Analysis (sPLSDA) of the post-resuscitation plasma from humans (a) and rodents (b) compared with their respective controls. In both human and rat plasma, there is a distinct metabolic separation of post-resuscitation plasma from control plasma that is very similar between both species. (CON: control, CA: post-CA and resuscitation).

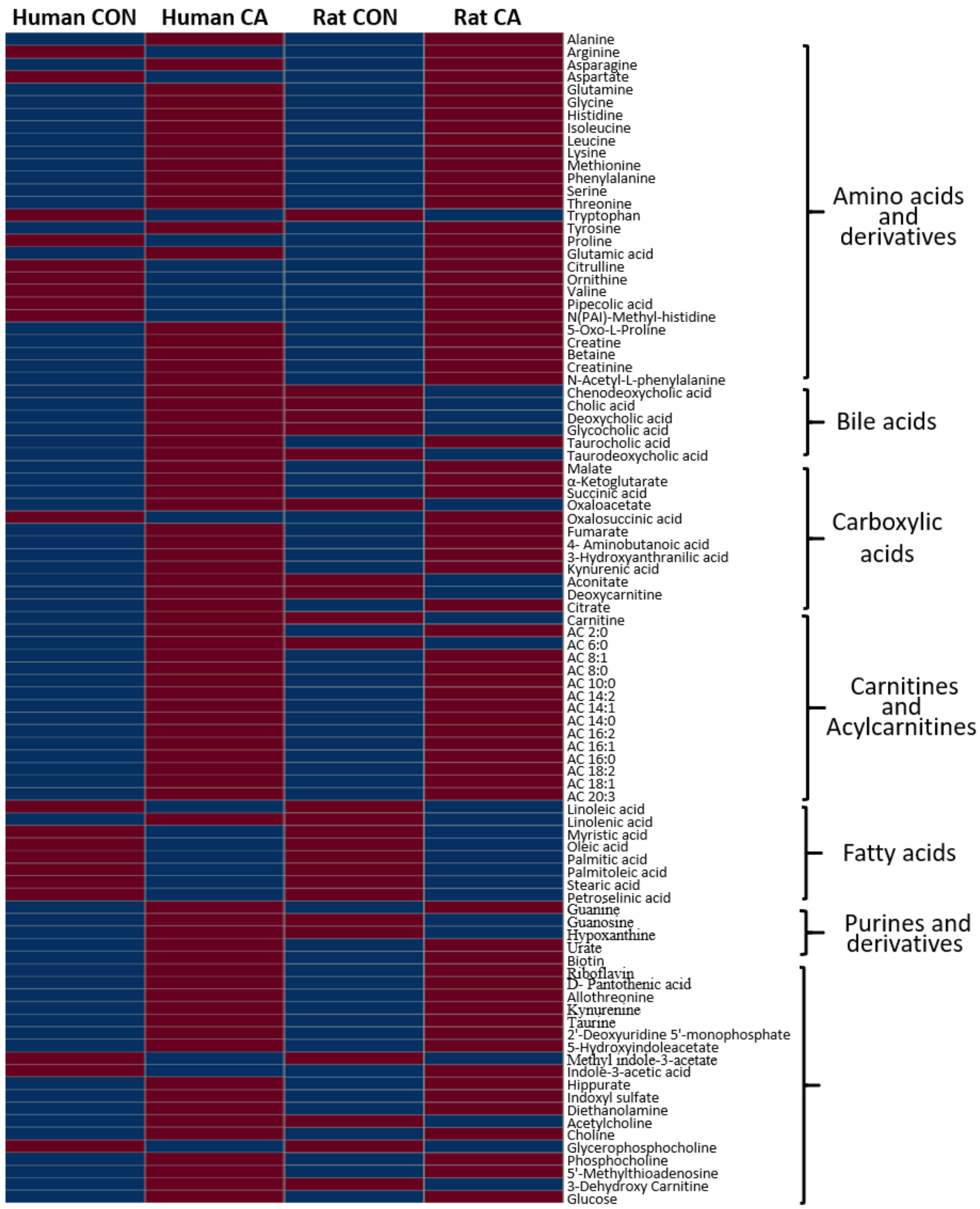

Supplementary Fig. 2. **Simplified heatmap depicting similar metabolic alterations in human and rat plasma after CA**. The mean metabolite values are represented. The colors of each metabolite correspond to the degree of variation after resuscitation with red indicating increased, while blue indicating decreased levels. Similar patterns emerge in both humans and rat. (CON: control, CA: post-CA and resuscitation).

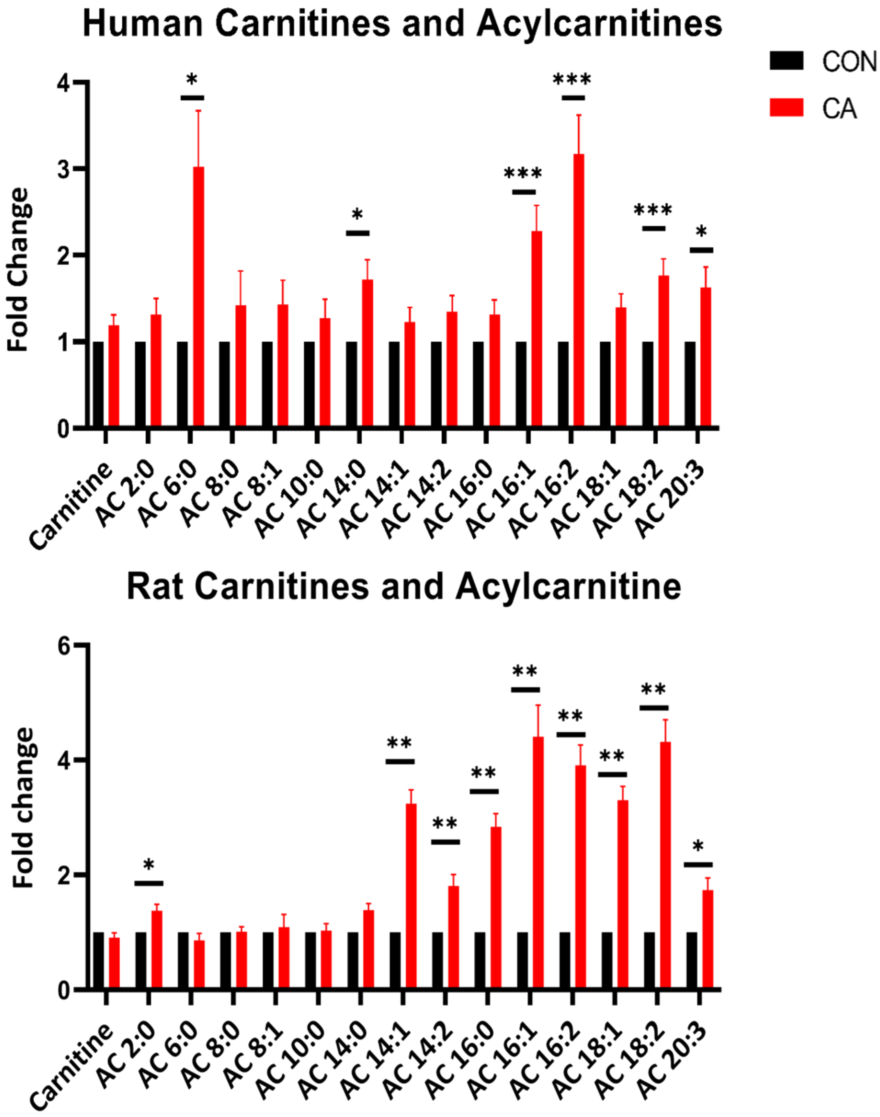

Supplementary Fig. 3: Longer-chain Acylcarnitines show similar alterations in humans and rats post-CA. Alterations in acylcarnitine species in human (top) and rat (bottom) plasma after CA and resuscitation. Both the rat and human plasma show an increase in the acylcarnitine species with longer fatty acid chains post-resuscitation except for AC 6:0 and AC 2:0 in humans and rats, respectively. (CON: control, CA: post-CA resuscitation human or rat, and AC: acylcarnitine). Data are displayed as mean ± SEM. Statistical analyses for each metabolite were performed using Mann-Whitney U test; *P<0.05, **P<0.01, and ***P<0.001.

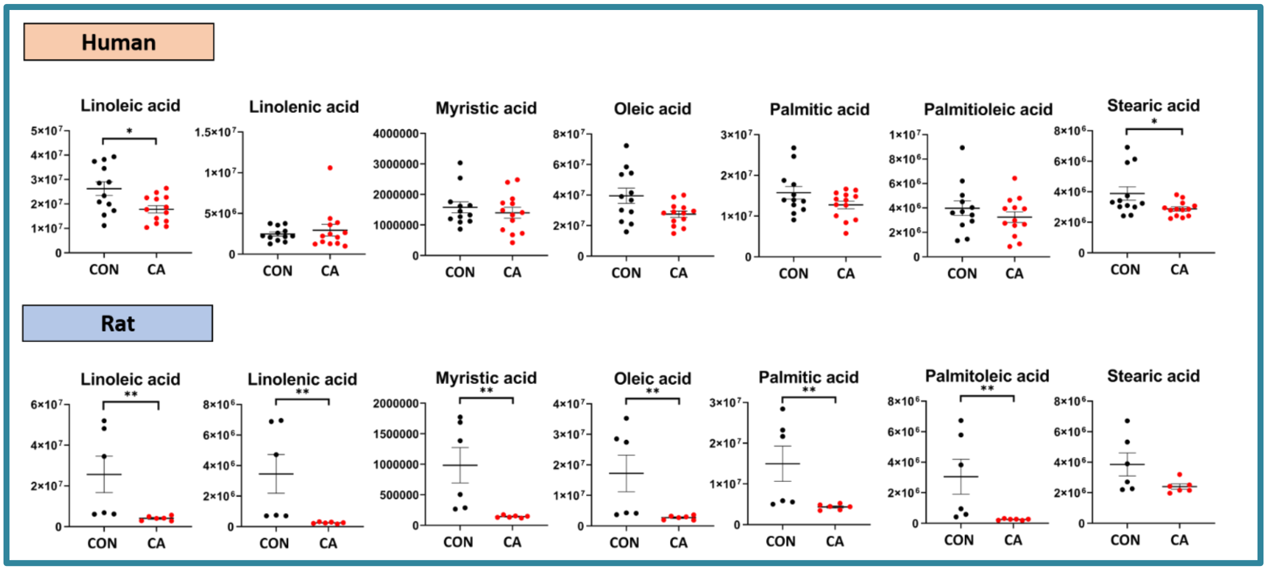

Supplementary Fig. 4: Similarly decreased free fatty acid levels in both humans and rats post-CA. Alterations in free fatty acids in human (top) and rat (bottom) plasma after CA and resuscitation. Both the rat and human plasma show a decrease in almost all free fatty acids, with more statistical significance observed in the rat plasma. Human plasma shows an overall decreasing trend. (CON: control, CA: post-CA resuscitation human or rat, and AC: acylcarnitine, y-axis; area under curve (AUC) of peak). Data are displayed as mean ± SEM. Statistical analyses for each metabolite were performed using Mann-Whitney U test; $*P<0.05$ and $**P<0.01$.
